# Role of amylopectin synthesis in *Toxoplasma gondii* and its implication in vaccine development against toxoplasmosis

**DOI:** 10.1098/rsob.200384

**Published:** 2021-06-16

**Authors:** Congcong Lyu, Xuke Yang, Jichao Yang, Lun Hou, Yanqin Zhou, Junlong Zhao, Bang Shen

**Affiliations:** ^1^ State Key Laboratory of Agricultural Microbiology, College of Veterinary Medicine, Huazhong Agricultural University, Wuhan 430070, People's Republic of China; ^2^ Key Laboratory of Preventive Medicine in Hubei Province, Huazhong Agricultural University, Wuhan 430070, People's Republic of China; ^3^ Hubei Cooperative Innovation Center for Sustainable Pig Production, Wuhan 430070, People's Republic of China

**Keywords:** chronic infection, bradyzoite, reactivation, starch synthase, CDPK2

## Abstract

*Toxoplasma gondii* is a ubiquitous pathogen infecting one-third of the global population. A significant fraction of toxoplasmosis cases is caused by reactivation of existing chronic infections. The encysted bradyzoites during chronic infection accumulate high levels of amylopectin that is barely present in fast-replicating tachyzoites. However, the physiological significance of amylopectin is not fully understood. Here, we identified a starch synthase (SS) that is required for amylopectin synthesis in *T. gondii*. Genetic ablation of SS abolished amylopectin production, reduced tachyzoite proliferation, and impaired the recrudescence of bradyzoites to tachyzoites. Disruption of the parasite Ca^2+^-dependent protein kinase 2 (CDPK2) was previously shown to cause massive amylopectin accumulation and bradyzoite death. Therefore, the *Δcdpk2* mutant is thought to be a vaccine candidate. Notably, deleting SS in a *Δcdpk2* mutant completely abolished starch accrual and restored cyst formation as well as virulence in mice. Together these results suggest that regulated amylopectin production is critical for the optimal growth, development and virulence of *Toxoplasma*. Not least, our data underscore a potential drawback of the *Δcdpk2* mutant as a vaccine candidate as it may regain full virulence by mutating amylopectin synthesis genes like SS.

## Graphical abstract

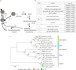

## 1. Background

*Toxoplasma gondii* is an obligate intracellular parasite of the protozoan phylum Apicomplexa that comprise many parasitic pathogens of medical and veterinary importance, such as *Plasmodium* and *Eimeria* species. Infections by *Toxoplasma* are highly prevalent in humans and animals [1,2]. One factor contributing to the wide spread of *T. gondii* is the multiple routes of transmission [3,4]. Oocysts shed by cats in the environment serve as a major source of infection to new hosts [5]. *Toxoplasma* can also be transmitted between intermediate hosts that include many warm-blooded animals [6–8]. For example, humans may get infected by consuming undercooked meat infected with *Toxoplasma* cysts [9]. *Toxoplasma gondii* exists in two asexual forms in its intermediate hosts. A fast-replicating tachyzoite form that underlies acute infection and associated clinical symptoms, and a slow-growing bradyzoite form that causes persistent, usually life-long, chronic infection [4,10]. Depending on the environmental cues, tachyzoites and bradyzoites can interconvert, which is crucial for the pathogenesis and transmission of *T. gondii* [11]. Nonetheless, the molecular mechanisms that govern such conversions remain largely elusive.

The encysted bradyzoites and oocysts contain abundant amylopectin granules, which are barely apparent in tachyzoites [12–16]. Such granules were first described over 50 years ago [17]; however, their metabolic pathways and biological significance are not yet well-examined experimentally. It is widely assumed that amylopectin is a rich source of energy for bradyzoites and oocysts, because they are enclosed by a thick wall that is poorly permeable to external nutrients [12]. It is also proposed that amylopectin may provide energy to bradyzoites and oocysts during their conversion to tachyzoites [18]. Recently, a CPDK2 in the parasite was shown to regulate the amylopectin production, likely through phosphorylation of vital enzymes involved in amylopectin synthesis and/or degradation [19,20]. The absence of CDPK2 causes tachyzoites and bradyzoites to accumulate massive amounts of amylopectin. As a probable consequence, the *Δcdpk2* mutants fail to produce tissue cysts [20]. Amylopectin accumulation in the *Δcdpk2* strain can be reversed by a point mutation (changing Ser 25 to Glu, which results in a hyperactive enzyme) in glycogen phosphorylase—an enzyme involved in amylopectin degradation [20]. These studies demonstrated the importance of amylopectin metabolism in parasites, though the exact physiological roles of amylopectin are still unclear. Our work focused on a starch synthase (SS) that we show is involved in amylopectin biosynthesis in *T. gondii*.

## 2. Methods

### 2.1. Parasite strains and growth *in vitro*

The ME49 strain of *T. gondii* and its derivative strains were propagated in human foreskin fibroblasts (ATCC no. SCRC-1041, USA), which were cultured in Dulbecco's modified Eagle's medium (DMEM) supplemented with 10% fetal bovine serum (Life Technologies, USA), 100 µg ml^−1^ streptomycin and 10 mM l-glutamine [21].

### 2.2. Construction of SS and CDPK2 mutants and phenotyping

Mutants lacking SS and/or CDPK2 were constructed by CRISPR/Cas9-assisted homologous gene replacement. Gene-specific CRISPR plasmids were generated by site-directed mutagenesis (using primers listed in electronic supplementary material, table S1), as previously described [22,23]. The donor DNA templates were constructed by cloning the 5′- and 3′-homology arms, flanking a drug selection marker (*DHFR* or *CAT*), into the pUC19 vector using the ClonExpress II One-Step cloning kit (Vazyme Biotech, China) [21]. The gene-specific CRISPR plasmids and homologous donor templates were co-transfected into extracellular tachyzoites of the ME49 or derivative strains, followed by selection with 1 µM pyrimethamine (Sigma-Aldrich, USA) or 30 µM chloramphenicol (Sigma-Aldrich, USA), and parasite cloning by limiting dilution. The positive mutant clones were identified by diagnostic PCRs (PCR1–3, using primers in electronic supplementary material, table S1). Plaque, replication and bradyzoite differentiation assays were performed using protocols reported earlier [24,25].

### 2.3. Western blotting

To generate antibodies against SS, a 6xHis-tagged polypeptide corresponding to the SS fragment from E136 to L587 was expressed and purified from *E. coli*. The recombinant protein was used to immunize rabbits for the production of polyclonal antibodies, which were tested by Western blotting as previously described [26]. Briefly, about 4 × 10^7^ parasites were lysed in 40 µl of SDS-sample buffer, of which 20 µl was loaded and resolved by 4–12% gradient SDS-PAGE gels, followed by protein transfer to nitrocellulose membranes and immunoblotting with rabbit anti-SS sera. The rabbit anti-*Tg*ALD antibody (provided by Dr David Sibley, Washington University in St Louis) was included as a loading control. Primary antibodies were detected by HRP conjugated goat anti-rabbit IgG (Boster Biological Technology, China). The blots were then developed by the ECL kit (Thermo Fisher Scientific, USA) and subsequently scanned by the Amersham Typhoon NIR imager (GE Healthcare, USA).

### 2.4. Periodic acid–Schiff staining

To stain tachyzoites, extracellular parasites were used to infect fresh HFF monolayers seeded on glass coverslips and cultured for 24 h. Then the samples were stained with periodic acid–Schiff (PAS) [26]. To stain bradyzoites, parasite cultures were subjected to alkaline stress (culture medium with pH = 8.2, ambient CO_2_) for 5 days in T25 flasks. Subsequently, syringe-released parasites were used to infect fresh HFF cells on coverslips and cultured under the same alkaline conditions for 4 days prior to staining [19]. All samples were fixed with 4% paraformaldehyde, permeabilized with Triton X-100 (Sigma-Aldrich, USA) and stained with Hoechst 33342 (Beyotime, China) and/or FITC-conjugated *Dolichos biflorus* agglutinin (DBA-FITC) (Vector Laboratories, USA). Successive PAS staining was performed by a standard procedure reported previously [19]. Briefly, samples were incubated in 1% periodic acid (Sigma-Aldrich, USA) for 5 min, washed with tap water for 1 min, and rinsed once with distilled water. Cells were then incubated with Schiff's reagent (Sigma-Aldrich, USA) for 15 min, washed with tap water for 10 min, and rinsed three times with PBS. Cultures were imaged by the Olympus BX53 microscope (Olympus, Japan) equipped with an Axiocam 503 mono camera (Carl Zeiss, Germany).

### 2.5. Metabolic labelling

Fresh extracellular tachyzoites (3 × 10^7^) were incubated in DMEM medium containing 8 mM ^13^C_6_-glucose (37°C, 4 h). Subsequently, the parasites were washed three times with glucose-free DMEM and resuspended in 50% aqueous methanol. Metabolites were extracted using established protocols and analysed by UHPLC-HRMS (ultra-high-performance liquid chromatography high-resolution mass spectrometry) [26]. Chromatographic separation was performed on a UHPLC system (Thermo-Fisher Ultimate 3000, Thermo Fisher Scientific, USA) with a Waters BEH Amide column (2.1 × 100 mm, 1.7 µm). The injection volume was 5 µl and the flow rate was adjusted to 0.35 ml min^−1^ with a linear gradient elution. The mobile phases consisted of water (phase A) and acetonitrile/water (90 : 10, v/v) (phase B). Both phases contained 10 mM ammonium formate (pH = 9.0). The eluents were analysed on a mass spectrometer (Thermo-Fisher Q Exactive Hybrid Quadrupole-Orbitrap) using the HESI (heated electro spray ionization) negative mode. A high-resolution scan was obtained (70–400 m/z) with AGC (automatic gain control) target set as 3 × 10^6^. The *m*/*z* spectra and relative abundance of metabolites were analysed by Xcalibur (v.4.0.27.19) (Thermo Fisher Scientific, USA).

### 2.6. Virulence tests and counting of parasite cysts in mice brains

Female ICR mice (7 weeks old) were subjected to intraperitoneal injection with purified tachyzoites of indicated strains (100 parasites/mouse), and animals were subsequently monitored daily for 30 days. An indirect ELISA test that detects TgMIC3 specific antibodies was used to determine the infection status of the surviving mice. Seropositive mice were anaesthetized and sacrificed to isolate the brain tissues, which were then homogenized and stained with DBA-FITC to determine the number and size of *Toxoplasma* cysts, as described [27]. All animal experiments were approved by the Scientific Ethics Committee of Huazhong Agricultural University (permit no. HZAUMO-2018-034).

### 2.7. Phylogenetic analysis

Protein sequences were retrieved from NCBI (https://www.ncbi.nlm.nih.gov). Sequences were aligned by Clustal W and curated to remove low-homology regions. The phylogenetic tree was constructed using the maximum-likelihood method based on the JTT matrix-based model in MEGA 7.0. Bootstrap analysis was performed with 1000 replicates. Finally, a tree was drawn to scale, with branch lengths measuring the number of substitutions per site. Sequences used include: *Toxoplasma gondii* starch synthase, XP_018638322.1; *Cystoisospora suis* starch synthase, PHJ19571.1; *Eimeria maxima* starch synthase, XP_013337552.1; *Neospora caninum* starch synthase, XP_003880146.1; *Cryptosporidium muris,* XP_002139398.1; *Galdieria sulphuraria* starch synthase, XP_005707965.1; *Porphyridium purpureum* glycogen synthase, KAA8498177.1; *Chondrus crispus* Starch synthase, XP_005718355.1; *Arabidopsis thaliana* starch synthase 1, OAO95110.1; *Oryza sativa* soluble starch synthase 1, AEB33739.1; *Triticum aestivum* starch synthase, AAB17085.1; *Solanum tuberosum* granule-bound starch synthase 1, NP_001274918.1; *Zea mays* granule-bound starch synthase 1, AQL02320.1; *Homo sapiens* glycogen synthase, AAB30886.1; *Danio rerio* glycogen synthase, NP_001018199.1; *Python bivittatus* glycogen synthase, XP_007427449.1; *Gallus gallus* glycogen synthase, XP_004938048.1.

### 2.8. Statistical analysis

Statistical analyses were performed in Prism 7 (GraphPad Software, USA), using Student's *t*-test, one-way ANOVA, two-way ANOVA or Gehan–Breslow–Wilcoxon test, as indicated in pertinent figure legends.

## 3. Results

### 3.1. Construction of a starch synthase deficient mutant

**Figure 1.**
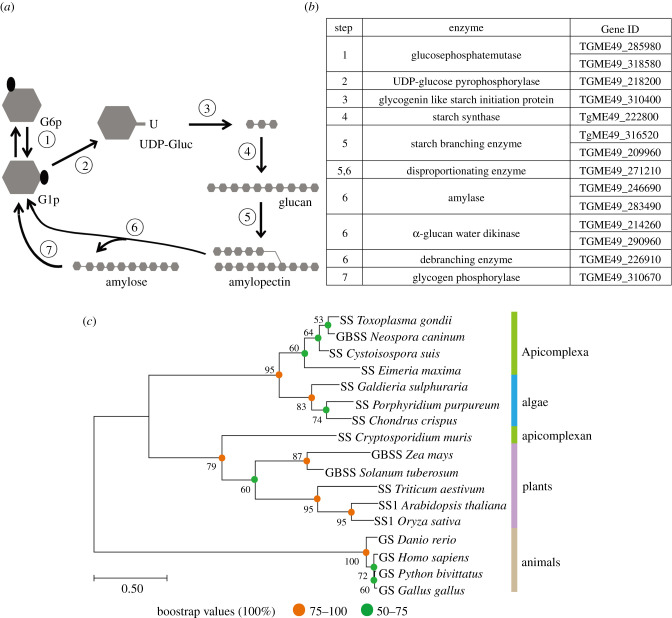
Enzymes involved in amylopectin metabolism in *T. gondii* based on *in silico* analysis*.* (*a)* Pathways of amylopectin synthesis and degradation. (*b*) Proteins mediating each reaction in (*a*) and their corresponding gene IDs. (*c*) Phylogenetic relationships of SS homologs from selected organisms. Protein sequences were retrieved from NCBI and the phylogenetic tree was constructed using the maximum-likelihood method (MEGA7).

We first searched for the enzymatic pathways that may catalyse amylopectin synthesis and degradation in *T. gondii*. The enzymes involved in starch metabolism in *Arabidopsis* and glycogen metabolism in humans were used as baits, to identify homologous proteins in the *Toxoplasma* genome. Hits from Blast searches and sequence analyses were used to construct a putative network involved in amylopectin metabolism in *T. gondii* (figure 1*a*,*b*). Our predicted pathways were similar to what has been proposed before [19], although small differences do exist. For example, we predicted that the gene TgME49_271210 encoded a disproportionating enzyme, which may contribute to both the synthesis and degradation of amylopectin. Whereas others suggested that it is a debranching enzyme involved in amylopectin degradation [19].

We focused on SS, which catalyses the elongation of α-1,4-linked glucan chains by adding a glucose unit from ADP-glucose or UDP-glucose to the non-reducing end (where glucose additions or removals occur in a glucan) (figure 1*a*). The *Toxoplasma* genome encodes a single SS (figure 1*b*). Based on our bioinformatic predictions, we assumed that it is likely the only enzyme to catalyse the elongation of glucose polymers during amylopectin synthesis in *T. gondii*. SS is well conserved in selected coccidia parasites, such as *Neospora* and *Eimeria* (figure 1*c*), which are known to harbor polysaccharide granules [28,29]. On the other hand, SS was not found in haematozoa (e.g. *Plasmodium*, *Babesia* and *Theileria*) that do not accumulate amylopectin. Except for the *Cryptosporidium* SS that is clustered with plant SS, other apicomplexan SS proteins are more closely related to corresponding enzymes in algae than those in plants or animals (figure 1*c*; electronic supplementary material, figure S1).

**Figure 2.**
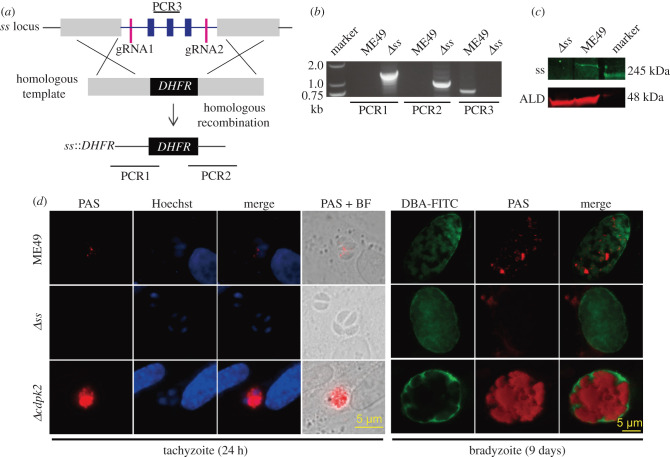
Amylopectin accumulation in the tachyzoites and bradyzoites of wild-type and mutant strains*.* (*a*) Genetic deletion of *SS* by double homologous recombination, mediated by a dual-*g*RNA CRISPR gene editing system. PCR1–3 are products of diagnostic PCRs used to identify the clonal mutants. (*b*) Diagnostic PCRs on a representative *Δss* clone. (*c*) Immunoblot confirming the loss of SS expression in the *Δss* mutant, ALD served as a protein loading control. (*d*) PAS staining of amylopectin in the parasites. Indicated strains were cultured under tachyzoite growth condition for 24 h, or under bradyzoite-inducing conditions (pH = 8.2, ambient CO_2_) for 9 days. Samples were subjected to DBA-FITC, PAS and Hoechst staining to visualize the cyst wall, amylopectin granules and nuclei, respectively.

To examine the physiological importance of SS in *T. gondii*, we ablated the *SS* gene in the type 2 strain ME49 by CRISPR/Cas9-mediated homologous gene replacement. Since the *SS locus* was relatively large (greater than 34 kb), a CRISPR plasmid containing two guide RNAs targeting the 5′ and 3′-ends was used (figure 2*a*). After transfection and drug selection, diagnostic PCRs were used to screen for the knockout parasite clones and *Δss* mutants were successfully obtained (figure 2*b*). To confirm the disruption of SS, we generated rabbit antisera and performed immunoblotting. In the parental ME49 strain, a protein band above 245-kDa was observed, which is consistent with the theoretical molecular weight of SS (330-kDa). This band was not detectable in the *Δss* mutant (figure 2*c*), suggesting successful deletion of SS.

### 3.2. Starch synthase is critical for amylopectin synthesis and its inactivation leads to reduced utilization of exogeneous glucose

To examine the role of SS during amylopectin synthesis, the *Δss* mutant was subjected to PAS staining of polysaccharides. Under standard growth condition, the ME49 tachyzoites were weakly stained, suggesting a mild accumulation of amylopectin. By contrast, no visible PAS signal was detected in the *Δss* mutant, indicating a lack of amylopectin. As a control, the *Δcdpk2* mutant was strongly stained, which was consistent with its high amylopectin accumulation [20] (figure 2*d*; electronic supplementary material, figure S2*a*). PAS staining on alkaline (pH = 8.2) induced bradyzoites was also performed. After 9 days of the pH-shift, most parasites were stained by DBA-FITC, a fluorescent dye that recognizes the cyst wall, confirming a successful induction of bradyzoites (figure 2*d*). Under alkaline conditions, the PAS staining signals in the ME49 parental strain were notably brighter than those in tachyzoites. Likewise, the *Δcdpk2* mutant exhibited massive amounts of amylopectin in alkaline-induced cultures. However, the *Δss* mutant did not show any PAS staining signal even under bradyzoite-inducing conditions (figure 2*d*; electronic supplementary material, figure S2*a*), further confirming that SS is needed for amylopectin synthesis in *Toxoplasma*. Amylopectin levels in ME49 and the *Δss* mutant were also determined by HPLC after being degraded to glucose by α-amylase and α-glucosidase. Although there was variation among experiments, our results repeatedly showed that the amylopectin level in the *Δss* mutant was lower than that in the parental strain ME49, at both the tachyzoite and the bradyzoite stages (electronic supplementary material, figure S2*b*).

**Figure 3.**
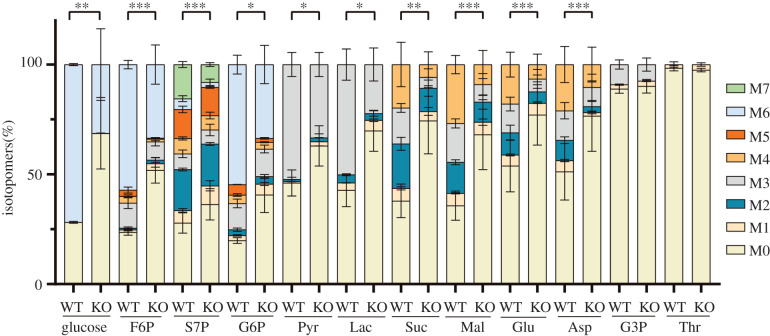
Utilization of exogenous glucose determined by metabolic tracing. Tachyzoites of the ME49 (WT) and *Δss* (KO) strains were incubated in DMEM medium containing 8 mM ^13^C_6_-glucose for 4 h, and incorporation of ^13^C into selected metabolites was determined by UHPLC-HRMS. Means ± s.e.m. of three independent experiments. *p ≤ 0.05, **p ≤ 0.01, ***p ≤ 0.001, two-way ANOVA. F6P, fructose-6-phosphate; S7P, sedoheptulose-7-phosphate; G6P, glucose-6-phosphate; Pyr, pyruvate; Lac, lactate; Suc, succinate; Mal, malate; Glu, glutamate; Asp, aspartate; G3P, glycerol-3-phosphate; Thr, threonine. M0–M7 denotes the number (0–7) of carbons labelled with ^13^C in the corresponding molecules.

We next investigated whether the SS deletion affected the catabolism of glucose, as it is the major substrate for amylopectin synthesis, as well as an important energy source for the parasites [30]. In this regard, we labelled the fresh extracellular parasites with ^13^C_6_-glucose for 4 h and measured the inclusion of isotopic carbon into glycolysis and TCA-cycle intermediates by mass spectrometry. Surprisingly, we observed that flux of ^13^C into glycolysis (glucose-6-phosphate, fructose-6-phosphate, pyruvate and lactate), TCA cycle (succinate and malate), pentose phosphate pathway (sedoheptulose-7-phosphate) and certain amino acids (glutamate and aspartate) was significantly decreased in the SS-knockout mutant when compared to the parental strain (figure 3). Moreover, much less cellular glucose was labelled with ^13^C in the mutant, suggesting reduced import of glucose into parasites from the medium. The reduced efficiency of glucose catabolism in the *Δss* mutant is consistent with its slower growth (see below). Although it is not clear whether reduced glucose catabolism is directly causing the growth defect of *Δss* mutant, these data indicate that robust uptake and utilization of exogenous glucose requires a functional SS. The fact that the *Δss* mutant was less efficient in using exogenous glucose prompted us to check whether it relied more on glutamine, another important carbon source for *Toxoplasma* parasites. In medium containing glucose but no glutamine, the *Δss* mutant had similar degree of growth reduction as that seen in medium containing both glucose and glutamine (electronic supplementary material, figure S3). Surprisingly, when both glucose and glutamine were taken away from the medium, the *Δss* mutant proliferated significantly faster than the parental strain ME49 (electronic supplementary material, figure S3). The underlying basis for the increased replication rate of the *Δss* mutant in the glucose and glutamine deficient medium is currently unknown, but it further suggests an altered metabolic capacity or need of this mutant.

### 3.3. Inactivation of starch synthase impairs the tachyzoite growth

**Figure 4.**
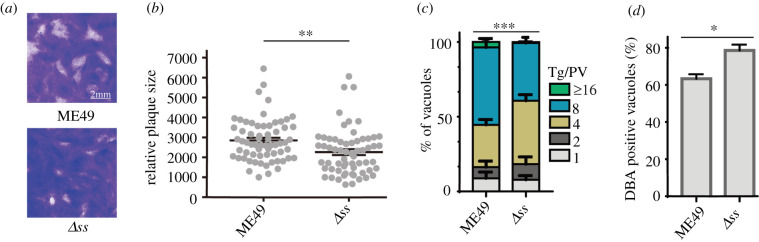
Growth and development of the Δss mutant *in vitro*. (*a*) Growth of the ME49 or *Δss* tachyzoites, as determined by plaque assays. Scale bar = 2 mm. (*b*) Relative size of the plaques formed by ME49 versus *Δss* strains cultured in HFF monolayers for 14 days. Means ± s.e.m., **p ≤ 0.01, Student's *t*-test. (*c*) Intracellular replication rates of the indicated strains, as determined by the fraction of vacuoles containing 1, 2, 4, 8 and 16 or more parasites. Means ± s.e.m. of three independent experiments, ***p ≤ 0.001, two-way ANOVA. (*d*) Bradyzoite differentiation rates of ME49 versus the *Δss* strains. Parasite cultures were induced in alkaline media for 4 days before DBA-FITC staining to determine the efficiency of bradyzoite transition. Means ± s.e.m. of three independent experiments, *p ≤ 0.05, Student's *t*-test.

In further experiments, we performed plaque assays in prolonged (two weeks) unperturbed cultures to examine the parasite fitness. The plaques formed by the *Δss* mutant were significantly smaller than those of the parental ME49 strain (figure 4*a,b*), while the number of plaques was indistinguishable, suggesting a slower growth of the mutant. Next, we tested the proliferation rate of the *Δss* mutant. In this regard, parasites were allowed to invade host cells for 1 h and then replicate for an additional 24 h. Subsequently, the number of parasite progeny in each parasitophorous vacuole (PV) was determined (figure 4*c*). Consistent with our plaque assays, the *Δss* mutant replicated significantly slower than the parental strain, as judged by fewer big vacuoles (with greater than eight tachyzoites) and more small vacuoles (less than four parasites). Our attempts to complement the *Δss* mutant were futile, likely due to the large size of this gene (genomic sequence greater than 34 kb). Nonetheless, we examined the phenotype of two additional independent *Δss* clones (electronic supplementary material, figure S4*a*,*b*), both of which exhibited defect in amylopectin production and growth as described above, affirming that the indicated phenotypes were caused by inactivation of SS.

### 3.4. Inability to synthesize amylopectin impairs the reactivation of bradyzoites to tachyzoites

**Figure 5.**
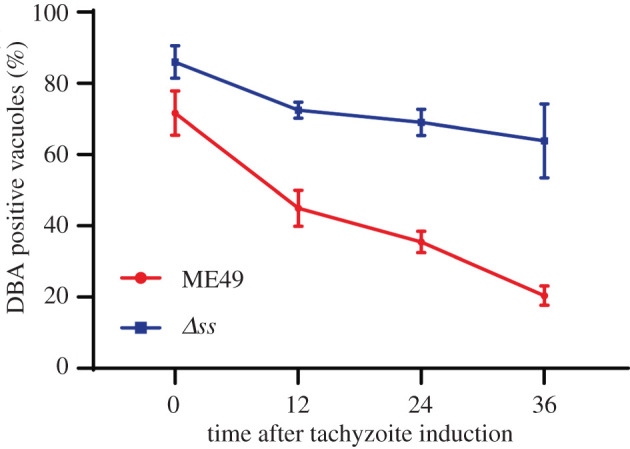
SS is important for the reactivation of bradyzoites *in vitro*. ME49 and *Δss* parasites were first cultured in the alkaline medium at ambient CO_2_ for 12 days to form bradyzoites (DBA-positive). The medium was then changed to favour tachyzoite growth at 5% CO_2_ for indicated periods, and samples were subjected to DBA-FITC staining. The decrease in DBA-positive vacuoles implies bradyzoite reactivation. Means ± s.e.m. of three independent experiments.

The fact that bradyzoites accumulate amylopectin granules and we observed a reduction of starch synthesis (see above) in the *Δss* mutant prompted us to test the role of SS during bradyzoite differentiation. We cultured the ME49 and *Δss* strains in alkaline medium (pH = 8.2) and ambient CO_2_ to induce bradyzoite formation, followed by DBA-FITC that stains the cyst wall. After 4 days induction, a modest but significant increase in the differentiation rate of all three independent *Δss* mutant clones (figure 4*d*; electronic supplementary material, figure S4*c*) was observed. For comparison, both strains had similar and very low levels of bradyzoite differentiation under normal growth conditions (pH = 7.2, 5% CO_2_), suggesting no significant spontaneous conversion of the *Δss* mutant. Because amylopectin may serve as an energy source during reactivation of bradyzoites into tachyzoites, we tested the hypothesis whether defects in amylopectin synthesis would impair the reactivation process using the *Δss* mutant. Cultures were incubated with alkaline medium and ambient CO_2_ for 12 days to enrich bradyzoites, and then the medium and growth conditions were reverted for normal tachyzoite cultivation to monitor the bradyzoite-to-tachyzoite conversion. As illustrated by DBA staining (figure 5), bradyzoites formed by the parental strain gradually switched to tachyzoites upon change to standard tachyzoite growth condition. Within 36 h, the fraction of DBA-positive vacuoles (indicative of bradyzoites) was reduced from 71.66 to 20.4%, in strong contrast to the *Δss* mutant, which displayed only a modest decline in cyst staining from 86 to 72.46% within the first 12 h and to 64% in 36 h) (figure 5). Reactivation was also assessed by the amount of time bradyzoite vacuoles took to egress. Both strains (ME49 and *Δss*) were first induced with alkaline for 12 days to form bradyzoites. Then the culture medium was changed to normal tachyzoite growth medium and the egress rates were monitored. Consistent with the DBA staining results (figure 5), the number of egressed vacuoles increased sharply within 36 h of tachyzoite induction in the ME49 strain and most vacuoles egressed by 48 h (electronic supplementary material, figure S5). By contrast, egress of the *Δss* vacuoles were delayed and only 50% of the vacuoles egressed even after 60 h of tachyzoite induction (electronic supplementary material, figure S5). Taken together, these data suggest a role of SS during the reactivation of bradyzoites to tachyzoites.

### 3.5. The starch synthase mutant displays heterogeneous tissue cysts *in vivo*

**Figure 6.**
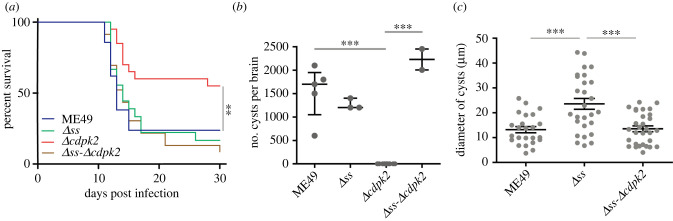
Virulence and cyst production of *T. gondii* mutants with altered amylopectin homeostasis. (*a*) Survival curves of mice infected with 100 tachyzoites of indicated strains (*n*=18 mice or more, **p ≤ 0.01, Gehan–Breslow–Wilcoxon test). (*b*) *Toxoplasma* cysts in the brain of animals that survived at day 30 in *a*. The number of cysts was determined by DBA-FITC staining of brain homogenates, Median with interquartile range, ***p ≤ 0.001, Student's *t*-test. (*c*) Relative size (diameter) of cysts detected in *b*, Means ± s.e.m. of three assays, ***p ≤ 0.001, Student's *t*-test.

To investigate the importance of amylopectin synthesis *in vivo*, we infected mice with the *Δss* mutant and parental strains and monitored the survival of infected animals. Despite a mild growth defect *in vitro*, the virulence of the *Δss* mutant was indistinguishable from that of the ME49 strain (figure 6*a*). We also examined the cyst burden in surviving animals (30 days post-infection). Surprisingly, although impaired in amylopectin synthesis, the *Δss* mutant produced a similar number of tissue cysts to the parental strain (figure 6*b*). However, the mean size (diameter) of the mutant cysts was higher and more heterogeneous (figure 6*c*), which suggests that amylopectin synthesis contributes to maintaining the regular size of tissue cysts.

### 3.6. SS deletion abolishes amylopectin accumulation in the *Δcdpk2* mutant and can restore its virulence and cyst formation

**Figure 7.**
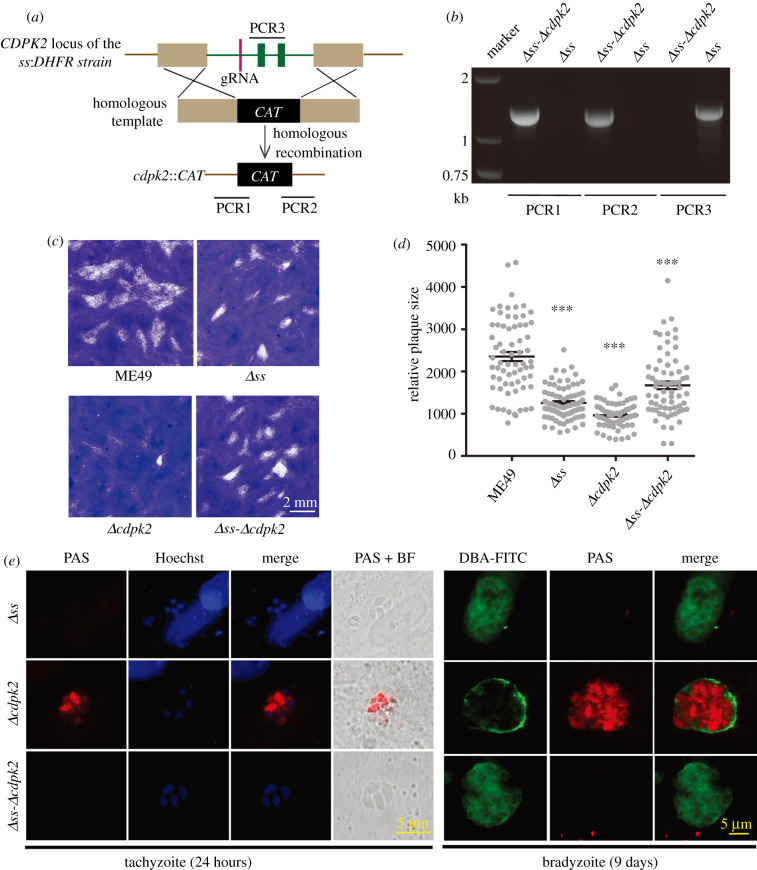
Construction and characterization of the Δ*ss*-Δ*cdpk2* double mutant. (*a*) Illustration of replacing the CDPK2 gene by a CAT selection marker in the *Δss* mutant. (*b*) Diagnostic PCRs of a representative clonal *Δss-Δcdpk2* mutant. (*c*) Growth of the shown strains, as determined by plaque assays. (*d*) Relative size of the parasite plaques formed in HFF monolayers. Means ± s.e.m., ***p ≤ 0.001, one-way ANOVA comparing the indicated strains to ME49. (*e*) Starch accumulation in the *Δss-Δcdpk2* mutant, as determined by PAS staining.

Previous work has shown that deletion of *CDPK2* in *T. gondii* leads to starch accumulation and is lethal to bradyzoites [20]. It is not clear however, whether the starch accrual underlies the observed phenotype. To address this question, we constructed the *Δcdpk2* and *Δss-Δcdpk2* mutants (figure 7*a*,*b*). Similar to the *Δss* strain, both *Δcdpk2* and *Δss-Δcdpk2* mutants displayed a modest but significant reduction in plaque size (figure 7*c*,*d*). PAS staining confirmed that *CDPK2* inactivation caused massive accumulation of amylopectin in tachyzoites as well as bradyzoites (figure 7*e*; electronic supplementary material, figure S2*a*), which was completely abolished upon deletion of *SS* (*Δss-Δcdpk2*), further confirming a role of SS in amylopectin synthesis.

Using our single and double mutants, we determined the importance of amylopectin synthesis during host infection. In the virulence test (figure 6*a*), the *Δcdpk2* mutant displayed a modest attenuation as judged by the higher survival rate (55%) of mice infected with this mutant than the parental ME49 strain (23.8%). Quite notably however, the *Δss-Δcdpk2* double mutant exhibited normal virulence (figure 6*a*). Moreover, consistent with the previous report [20], the mice that survived the infection by the *Δcdpk2* mutant did not harbour any cysts in their brain (figure 6*b*) [20]. However, the cyst formation in the *Δss-Δcdpk2* double mutant was similar to the parental strain. Similarly, the size distribution of the brain cysts derived from the *Δss-Δcdpk2* mutant was indistinguishable from that of the wild-type strain (figure 6*c*). In brief, our results show that SS disruption not only reversed the virulence defects of the *Δcdpk2* mutant but also restored its cyst formation capacity.

## 4. Discussion

Amylopectin granules are a hallmark of the bradyzoite and oocyst stages, which distinguish them from the fast-replicating tachyzoite stage of *T. gondii* [16]. In this study, we predicted the underlying metabolic pathways, and revealed that SS is essential for amylopectin production. Mutants lacking SS are unable to synthesize amylopectin in either tachyzoite or bradyzoite stages. Moreover, deletion of *SS* in the *Δcdpk2* strain completely reversed the amylopectin accumulation phenotype of the latter mutant, further confirming a role of SS in starch synthesis. The *Δss* mutant therefore allowed us to assess the importance of amylopectin in parasites. Our results demonstrate that SS is required for optimal growth of tachyzoites, likely by promoting the utilization of exogenous glucose. Importantly, we show that amylopectin synthesis is critical for the reactivation of bradyzoites to tachyzoites under favourable conditions. Mutants lacking *SS* were able to differentiate to bradyzoites *in vitro* as well as *in vivo*. At least in the *in vitro* model, bradyzoites of the *Δss* mutant did not respond to the reactivation signals effectively and were reluctant to convert to tachyzoites. This is the first genetic evidence to the best of our knowledge revealing that amylopectin may contribute to the reactivation of chronic toxoplasmosis.

Amylopectin accumulates in selected life cycle stages of coccidian parasites. Its physiological function has never been fully defined however. Early studies have shown that treating *Eimeria* or *Cryptosporidium* oocysts with high temperature (35°C or above) gradually depletes amylopectin and decreases the infectivity and durability of oocysts [31,32]. It was proposed that amylopectin may serve as a reservoir of energy in oocysts or tissue cysts. This hypothesis has never been tested by genetic approaches. Our *T. gondii Δss* mutant defective in amylopectin synthesis offered an opportunity to test this. While the *Δss* mutant was able to form bradyzoites upon induction with stress conditions, its bradyzoite to tachyzoite conversion was impaired after changing the cultures back to tachyzoite growth conditions. We still do not understand how exactly SS and amylopectin help recrudescence. The metabolic tracing experiments indicate that even in tachyzoites, which do not accumulate large amounts of amylopectin, *SS* deletion reduced the uptake and utilization of exogenous glucose (figure 3). These data suggest that some of the imported glucose is converted to glucans and then degraded to enter glycolysis and TCA cycle in tachyzoites. A dynamic amylopectin synthesis and breakdown may allow the parasite to quickly adapt to changing environments. It is plausible that reactivation of mature cysts needs a robust energy supply and catabolism of amylopectin is a critical contributor, which may be more difficult to achieve through exogenous glucose in bradyzoites when compared to tachyzoites.

Previous work has demonstrated that *CDPK2* inactivation caused over-accumulation of amylopectin in tachyzoites and bradyzoites [20]. The *Δcdpk2* mutant displays a reduced virulence and does not form cysts *in vivo*, although it is not clear whether abnormal amylopectin accumulation is directly responsible for these defects. Our results showed that *SS* deletion abolished amylopectin accumulation and restored the virulence and cyst formation defects in the *Δcdpk2* mutant, revealing that those deficiencies are indeed caused by the overly accumulated starch. Due to a modest attenuation of virulence and lack of cyst formation, the mutants lacking CDPK2 were proposed as a live vaccine candidate [33]. However, our work raises safety concerns to this vaccine candidate, as the *Δcdpk2* mutants can readily regain normal virulence and cyst formation capacity by inactivating genes like *SS* involved in SS.

## Data accessibility

This article has no additional data.
